# VEGFR and DPP-IV as Markers of Severe COVID-19 and Predictors of ICU Admission

**DOI:** 10.3390/ijms242317003

**Published:** 2023-11-30

**Authors:** Ewa Pius-Sadowska, Piotr Kulig, Anna Niedźwiedź, Bartłomiej Baumert, Karolina Łuczkowska, Dorota Rogińska, Anna Sobuś, Zofia Ulańczyk, Miłosz Kawa, Edyta Paczkowska, Miłosz Parczewski, Anna Machalińska, Bogusław Machaliński

**Affiliations:** 1 Department of General Pathology, Pomeranian Medical University in Szczecin, Al. Powstańców Wielkopolskich 72, 70-111 Szczecin, Poland; piotrkulig@interia.eu (P.K.); ania.niedzwiedz@gmail.com (A.N.); bartlomiej.baumert@pum.edu.pl (B.B.); karolina.luczkowska@pum.edu.pl (K.Ł.); dorota.roginska@pum.edu.pl (D.R.); anna.sobus@pum.edu.pl (A.S.); zofia.ulanczyk@pum.edu.pl (Z.U.); edyta.paczkowska@pum.edu.pl (E.P.); 2 Department of Infectious, Tropical Diseases and Immune Deficiency, Pomeranian Medical University in Szczecin, Arkońska 4 Street, 71-455 Szczecin, Poland; milosz.parczewski@pum.edu.pl; 3 First Department of Ophthalmology, Pomeranian Medical University, Al. Powstańców Wielkopolskich 72, 70-111 Szczecin, Poland; anna.machalinska@pum.edu.pl

**Keywords:** severe COVID-19, VEGFR, DPP-IV, angiogenesis, hypercoagulation

## Abstract

The pathophysiology of the severe course of COVID-19 is multifactorial and not entirely elucidated. However, it is well known that the hyperinflammatory response and cytokine storm are paramount events leading to further complications. In this paper, we investigated the vascular response in the pathophysiology of severe COVID-19 and aimed to identify novel biomarkers predictive of ICU admission. The study group consisted of 210 patients diagnosed with COVID-19 (age range: 18–93; mean ± SD: 57.78 ± 14.16), while the control group consisted of 80 healthy individuals. We assessed the plasma concentrations of various vascular factors using the Luminex technique. Then, we isolated RNA from blood mononuclear cells and performed a bioinformatics analysis investigating various processes related to vascular response, inflammation and angiogenesis. Our results confirmed that severe COVID-19 is associated with vWF/ADAMTS 13 imbalance. High plasma concentrations of VEGFR and low DPP-IV may be potential predictors of ICU admission. SARS-CoV-2 infection impairs angiogenesis, hinders the generation of nitric oxide, and thus impedes vasodilation. The hypercoagulable state develops mainly in the early stages of the disease, which may contribute to the well-established complications of COVID-19.

## 1. Introduction

The first infection with the novel coronavirus named SARS-CoV-2 was reported and confirmed in the Chinese city of Wuhan in December 2019 [1]. Shortly thereafter, the virus spread all over the world and became a great challenge for governments and healthcare systems worldwide. Multiple variants of the virus were identified throughout the pandemic. In particular, the Delta variant was highly associated with severe disease [2,3], whereas others such as Omicron, although highly contagious [4], typically caused clinically mild infections [5]. Currently, the most prevalent SARS-CoV-2 strain is EG.5 (Eris)—a subvariant of Omicron [6]. The symptomatology of COVID-19 disease is very diverse. Initial symptoms are typical of a respiratory infection and include fever, cough, and dyspnea, as well as fatigue and malaise. Moreover, gastrointestinal symptoms such as diarrhea, have also been reported [7]. Neurological manifestations such as anosmia and ageusia are believed to be characteristic of COVID-19, especially at the initial stages of the disease [8]. As the disease progresses in some patients, the signs and symptoms of pneumonia may contribute to the clinical manifestation [9]. While the vast majority of cases are mild, there is a small proportion of individuals who suffer from a severe form of the disease. They require admission to hospital or even intensive care unit (ICU). Furthermore, they may require non-invasive or invasive ventilation, alongside antipyretics, antiviral agents, antibiotics as well as steroids. Selected and particularly complex cases may require treatment by drugs mediating the immune system and even plasma exchange therapy [9]. Due to its high contagiousness, the absolute number of individuals who experienced a severe course of COVID-19 was high. Cumulative estimates of severity and mortality are established to be 18.0% and 3.2%, respectively [10]. In addition to immediate complications, people who contract SARS-CoV-2 infections may experience numerous long-term consequences affecting many organs and systems, which are usually referred to as “long COVID”. Clinical manifestations are diverse and include neurological symptoms such as fatigue, memory impairment, palpitations, cough and shortness of breath, and the development of diabetes, autoimmune diseases and reproductive system dysfunction [11,12].

The pathophysiology of severe COVID-19 is multifactorial and is associated with cytokine storm and hyperinflammatory responses [13]. There are several serum biomarkers associated with severe disease. One of the most important and best known is IL-6, which is a crucial proinflammatory cytokine, although its pleiotropic properties reach far beyond the simple promotion of inflammatory response [14]. With regards to COVID-19, it was demonstrated that serum concentration of this molecule is positively related to the severity of the infection. On top of that, its concentrations were shown to be associated with clinical outcome [15,16]. Moreover, IL-6 not only can be considered as a serum biomarker of serious clinical outcome, but may also play a causative role in the pathophysiology. Tocilizumab, a monoclonal antibody against the IL-6 receptor, has been shown to improve outcomes in critically ill individuals [17,18,19,20]. This implies that IL-6 has a critical role in the pathophysiology of severe COVID-19.

In addition to the hyperinflammatory reaction overcoming the adaptive potential of human organisms, the involvement of blood vessels, especially the microcirculation, seems to be a critical aspect in the pathophysiology of COVID-19. Particularly in the severe course of the disease, which is frequently associated with acute respiratory distress syndrome (ARDS). Joffre and colleagues investigated how the infection affects small blood vessels. Their results revealed that the virus has the ability to penetrate and replicate in endothelial cells. The replicating virus, but not spike protein and dead pathogen, increases endothelial permeability and secretion of plasminogen activator inhibitor 1 (PAH-1) and vascular endothelial growth factor (VEGF). Furthermore, they revealed that serum levels of endothelial activation and biomarkers of damage were increased in COVID-19 patients and correlated with disease severity [21]. Another hallmark of COVID-19 vasculopathy is a hypercoagulable state, endothelial activation, and vasoconstriction associated with nitric oxide deficiency [22]. 

We also investigated the pathophysiology and mechanisms underlying severe manifestation of COVID-19. We have demonstrated that ICU admission is associated with elevated plasma CXCL8 and CCL2 concentrations. Interestingly, expression levels of these cytokines, exemplified by mRNA levels, were low. Moreover, cytomegalovirus (CMV) seropositivity also correlated with the severe course of the disease [23]. While much has been discovered, for instance, the critical role of IL-6, there are aspects of severe COVID-19 that require further research. Therefore, we decided to investigate less explored aspects of severe SARS-CoV-2 infection. In this paper, we investigated the vascular response in the pathophysiology of severe COVID-19. We analyzed and compared the concentrations of various vasoactive factors in the plasma of SARS-CoV-2-positive individuals admitted to the ICU and those with only mild symptoms. In addition, we isolated the genetic material from blood mononuclear cells and conducted in-depth studies to identify new pathophysiological factors determining the clinical outcomes of COVID-19 patients. 

## 2. Results

### 2.1. Comparison of Plasma Concentrations of Selected Vasoactive Agents between ICU and Non-ICU Patients

In the first step of our analysis, we investigated differences in plasma levels of various vascular factors between the ICU and the non-ICU group. We included the following molecules in our analysis: von Willebrand factor (vWF), Serpin E1, E-selectin, thrombomodulin, epithelial growth factor (EGF), Angiopoetin-2, Endothelin-1, fibroblast growth factor (FGF-1), vascular endothelial growth factor receptor 1 (VEGFR1), vascular endothelial growth factor (VEGF), platelet-derived growth factor AA (PDGF-AA), platelet-derived growth factor AB (PDGF-AB), ADAMTS13, fms like tyrosine kinase 3 (FLT-3) and dipeptidyl peptidase-4 (DPPIV). The selection was based upon commercially available kits. 

**Figure 1:**
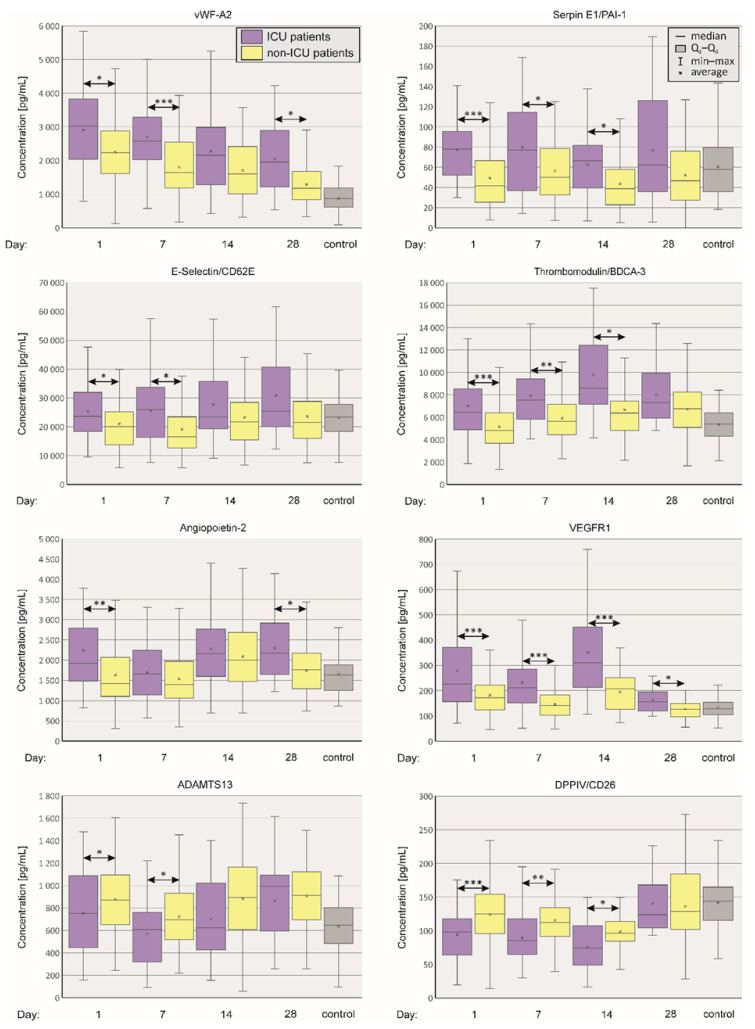
Boxplots showing plasma vascular factor levels in patients depending on the severity of the course of COVID-19 and in SARS-CoV2-negative controls. Figure depicts vascular factors which were highly associated with the severe disease. Mann–Whitney U test, *p* value < 0.05 was considered statistically significant. *p* < 0.05—*, *p* < 0.001—**, *p* < 0.0001—***.

Figure 1 depicts plasma levels of vasoactive agents which were highly associated with the severe disease and thus of clinical importance. Plasma levels of vWF and E-selectin were increased until day 7 of infection, while Serpin E1 and thrombomodulin were elevated until day 14.

Plasma levels of Endothelin-1 were significantly elevated in non-ICU patients on day 7, while concentrations of Angiopoietin-2 were markedly increased in the ICU group on day 1. Concentration of VEGFR1 was significantly increased in the ICU group on days 1, 7, 14 and 28. The analysis revealed that the concentration of ADAMTS 13 was significantly increased in non-ICU patients on days 1 and 7 (thus severe disease is associated with diminished ADAMTS 13 level). FLT-3 concentration was significantly increased in ICU patients only on day 28. Plasma DPPIV was elevated in non-ICU patients on days 1, 7 and 14. 

**Figure 2:**
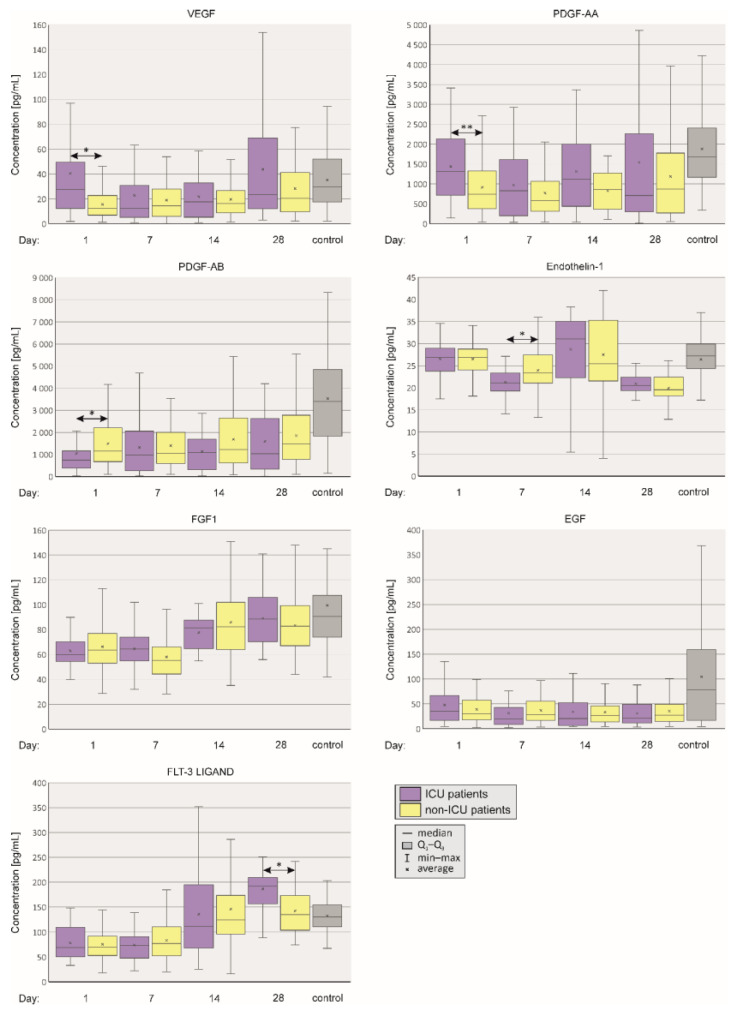
Boxplots showing plasma vascular factor levels in patients depending on the severity of the course of COVID-19 and in SARS-CoV2-negative controls. Figure depicts vascular factors which were not associated with the severity of the disease. Mann–Whitney U test, *p* value < 0.05 was considered statistically significant. *p* < 0.05—*, *p* < 0.001—**.

Figure 2 depicts plasma levels of vascular agents which were less associated with the severity of the disease. We did not detect any difference between SARS-CoV-2 positive individuals and healthy controls in plasma EGF and FGF1 levels. Moreover, plasma VEGF and PDGF-AA concentrations were significantly higher in the ICU group only on day 1, while PDGF-AB concentration was significantly higher on day 1 in the non-ICU group. FLT-3 concentration was significantly increased in ICU patients only on day 28. There were no statistically significant differences in concentrations of FGF-1 between the groups. 

In summary, we demonstrated that severe COVID-19 is associated with ADAMTS 13/vWF imbalance. Furthermore, severe COVID-19 is associated with high plasma concentrations of VEGFR and low DPP-IV, which can be considered as potential predictors of ICU admission. 

### 2.2. Blood Coagulation, Platelet Activation and Aggregation 

In the next step, we conducted an in-depth bioinformatics analysis investigating the activity of processes related to blood coagulation, platelet function, angiogenesis and the action of nitric oxide. 

**Figure 3:**
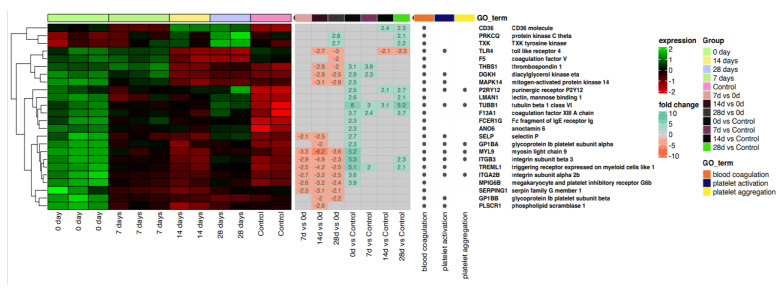
The bioinformatics analysis of genes and processes associated with blood coagulation. Heatmap with hierarchical clustering of differentially expressed genes (categorized according to gene ontology biological process terms—GO BP) in response to COVID-19-induced vascular stress in all analyzed groups (on days 0, 7, 14, 28 and control). Expression values are scaled by rows and presented as a color gradient ranging from red (low expression) to green (high expression). Log2 signal intensity values for any single gene were resized to Row Z-score scale (from −2, the lowest expression, to +2, the highest expression for a single gene). Gene symbols and gene names of differentially expressed genes were shown. Values (from −10 to +10) representing the average fold changes in gene expression between pairs of compared groups were also presented as a color gradient.

In general, the control group is characterized by low expression of the genes related to blood coagulation (Figure 3). On the other hand, in the study group (COVID-19 positive), hypercoagulation occurs on day 0, manifested by a peak in upregulation of coagulation, platelet activation and aggregation. In the subsequent days, these genes and processes tend to be downregulated. On day 14, the coagulation activity of the study group is comparable to the control group. A similar trend is visible on day 28.

### 2.3. Angiogenesis

**Figure 4:**
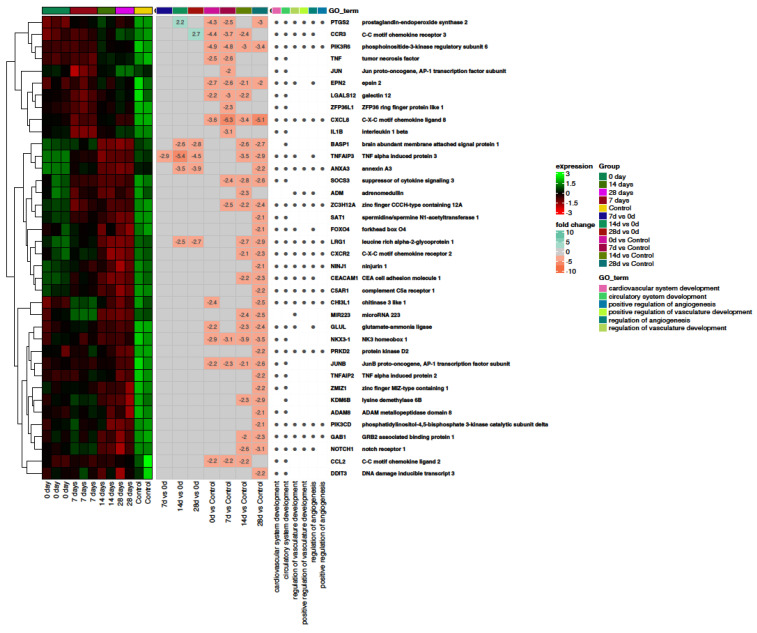
The bioinformatics analysis of genes and processes associated with angiogenesis. Heatmap with hierarchical clustering of differentially expressed genes (categorized according to gene ontology biological process terms—GO BP) in response to COVID-19-induced vascular stress in all analyzed groups (on days 0, 7, 14, 28 and control). Expression values are scaled by rows and presented as a color gradient ranging from red (low expression) to green (high expression). Log2 signal intensity values for any single gene were resized to Row Z-score scale (from −3, the lowest expression, to +3, the highest expression for a single gene). Gene symbols and gene names of differentially expressed genes were shown. Values (from −10 to +10) representing the average fold changes in gene expression between pairs of compared groups were also presented as a color gradient.

Figure 4 presents a heatmap with hierarchical clustering of differentially expressed genes related to the regulation of angiogenesis. Overall, they are upregulated in the control group and downregulated in the COVID-19 positive cohort. This implies that COVID-19 is associated with inhibition of angiogenesis.

### 2.4. Nitric Oxide

**Figure 5:**
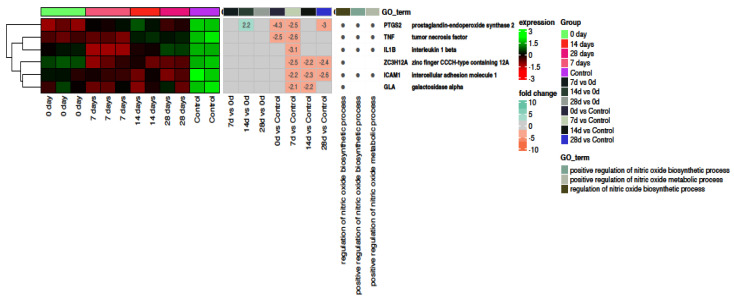
The bioinformatic analysis of genes and processes associated with nitric oxide-related actions. Heatmap with hierarchical clustering of differentially expressed genes (categorized according to gene ontology biological process terms—GO BP) in response to COVID-19-induced vascular stress in all analyzed groups (on days 0, 7, 14, 28 and control). Expression values are scaled by rows and presented as a color gradient ranging from red (low expression) to green (high expression). Log2 signal intensity values for any single gene were resized to Row Z-score scale (from −3, the lowest expression, to +3, the highest expression for a single gene). Gene symbols and gene names of differentially expressed genes were shown. Values (from −10 to +10) representing the average fold changes in gene expression between pairs of compared groups were also presented as a color gradient.

Figure 5 demonstrates a heatmap with hierarchical clustering of differentially expressed genes related to the regulation of nitric oxide biosynthesis and metabolic processes. All nitric oxide-related actions were upregulated in the control group compared to the study group at all time points.

To sum up, bioinformatics analysis revealed that the hypercoagulable state is developed predominantly in the initial phase of SARS-CoV-2 infection.

## 3. Discussion

The pathophysiology of severe COVID-19 is multifactorial, still not entirely explained, and is therefore the subject of ongoing research. There were multiple identified risk factors for a severe disease course. Based on the clinical characteristics of the patients and their medical history, a subpopulation of individuals who are particularly prone to ICU admission can be identified. For instance, it is very well-established that age, obesity, smoking, and comorbidities such as cardiovascular disorders and chronic diseases affecting respiratory system or cancer are risk factors for severe SARS-CoV-2 infection [24,25,26,27,28,29]. In addition to the clinical characteristic of the patients, molecular markers of worse clinical outcomes have been identified. For example, according to a study conducted by Sieńko and colleagues, ACE2 polymorphisms affect the severity of COVID-19 disease independently of other well-established risk factors [30]. While our team found that CMV seropositivity, elevated plasma CXCL8 and CCL2 concentrations are predictors of ICU admission [23]. The role of pro-inflammatory cytokines such as IL-6 is very well understood [15,16]. Despite the complexity of the interactions leading to severe disease, ultimately all mechanisms contribute to the cytokine storm and hyper-inflammatory response [13].

In this paper, we investigated the vascular response in the pathophysiology of severe COVID-19. We analyzed and compared the concentrations of various vasoactive factors in the plasma of SARS-CoV-2 positive individuals admitted to the ICU and those with only mild symptoms. In addition, we isolated the genetic material from the blood mononuclear cells and conducted in-depth studies to identify new pathophysiological factors determining the clinical outcomes of COVID-19 patients. We collected samples and analyzed data from patients infected with Delta variant due to its clinical relevance i.e., patients infected with Delta variant were more likely to succumb to severe disease [2,3].

First, we demonstrated that plasma levels of vWF and E-selectin were elevated in the ICU group during the first week of hospitalization. In addition, Serpin E1 and thrombomodulin concentrations remained elevated in severely ill patients for longer, up to day 14. The role of vWF seems particularly important. The interplay between vWF and ADAMTS13 are crucial in the pathogenesis of thrombotic microangiopathy such as thrombotic thrombocytopenic purpura (TTP) [31]. In addition, an imbalance between vWF and ADAMTS-13 i.e., elevated levels of vWF and decreased levels of ADAMTS13, has been shown to cause ARDS associated with COVID-19. Moreover, plasma exchange restores the proper vWF/ADAMTS13 ratio, reduces inflammation and benefits ventilation parameters [32]. Various studies reported that, in severe COVID-19, vWF levels tend to be elevated, accompanied by decreased ADAMTS13 levels [33,34,35,36]. This imbalance can promote thrombosis, contributing to various complications associated with COVID-19. Our study revealed similar findings regarding vWF/ADAMTS 13 ratio (see Figure 1). We also demonstrated that plasma levels of Serpin E1 (plasminogen activating inhibitor, PAI–1) and thrombomodulin were significantly elevated in the ICU group during first two weeks of the infection implying their association with the severe disease. PAI-1 is a crucial inhibitor of fibrinolysis [37]. Its increased plasma activity in patients suffering from a severe disease might additionally contribute to thrombotic complications and determine poor clinical outcome. Indeed, it was demonstrated that PAI-1 levels could independently predict disease severity and mortality rates for patients with COVID-19 [38]. On the contrary, thrombomodulin possess multiple anti-inflammatory and anticoagulatory properties [39]. Its increased levels in severely ill COVID-19 patients might an attempt to counteract increased proclivity to thrombosis in the severe disease. 

Subsequently, we found that elevated plasma VEGFR concentration is associated with severe course of the COVID-19 disease. VEGF concentration was also elevated in the ICU group, although a statistically significant difference was visible only on the first day. The VEGF/VEGFR axis is crucial in promoting both normal and pathological angiogenesis [40], and exhibits various pro-inflammatory properties [41,42]. In the severe course of COVID-19, microcirculatory disorders contribute to increased endothelial permeability, ultimately leading to ARDS. The exact pathophysiology of vascular damage in SARS-CoV-2 infection has been extensively studied. Several endothelial biomarkers contributing to vascular pathology have been identified, including VEGFR. Birnhuber et al. collected post-mortem lung tissue samples from 19 critically ill COVID-19 patients and 11 age- and sex-matched autopsy controls with no underlying lung pathology. Their results revealed an upregulation of genes encoding endothelial factors, including VEGFR2, in COVID-19 lung tissue compared to controls, suggesting their role in vascular pathology in severe disease [43]. Similarly, another post-mortem analysis of lung tissue obtained from critically ill COVID-19 patients showed overexpression of biomarkers involved in endothelial dysfunction, microthrombosis and angiogenesis, including VEGF and VEGFR. Furthermore, histopathology revealed microthrombosis, endothelial activation, and vascular hyperplasia, suggesting a role of endothelial dysfunction mediated by, among others, the VEGF/VEGFR axis in the pathogenesis of severe COVID-19 [44]. Another study identified several VEGFR polymorphisms that were associated with poorer clinical outcomes [45]. 

DPP-IV, also known as CD26, is expressed on a variety of cells, and is involved in multiple biological actions. Predominantly, it participates in the regulation of glucose metabolism [46]. DDP-IV inhibitors have been successfully implemented in the treatment of type 2 diabetes [47]. In addition, DPP-IV is involved in the immune response as it is expressed in different subpopulations of immune cells [48]. Moreover, it is believed to be a pro-inflammatory cytokine that promotes insulin resistance and metabolic syndrome [49]. Regarding COVID-19, DPP-IV has been shown to be involved in virus entry into host cells [50,51,52]. In our study, we demonstrated a statistically significant decrease in CD26 plasma concentration in the ICU group, predominantly during the first 14 days of the infection. Therefore, a decreased plasma DPP-IV/CD26 concentration can be considered a potential biomarker of severe COVID-19 and ICU admission. Schlicht et al. revealed that serum DPP-IV concentrations were markedly decreased in SARS-CoV-2 positive patients compared to healthy controls, but surprisingly did not differ between patients suffering from severe disease and controls [53]. Due to the hypothesis that DPP-IV may be involved in mediating viral entry and contributing to the severity of SARS-CoV-2 infection, DPP-IV inhibitors have been investigated in the COVID-19 management. There were several studies reporting the beneficial effects of DPP-IV inhibitors on COVID-19 in diabetic patients [54,55]. Moreover, a DPP-IV inhibitor as an add-on to standard therapy has been shown to improve clinical outcomes, radiological scores, and inflammatory biomarkers compared to standard therapy in non-diabetic patients with COVID-19 [56]. On the other hand, there have been studies that found otherwise and showed no beneficial effects of DPP-IV blockade [57,58]. Recent evidence suggests that DPP-IV and its inhibitors play a role in the pathogenesis of severe COVID-19 [59,60]. Nevertheless, this area requires further research to elucidate its exact role in mediating severe manifestation of the disease, especially when clinical trials investigating DPP-IV inhibitors in the COVID-19 management provide conflicting results.

Next, we conducted a thorough bioinformatics analysis and investigated the processes involved in hemostasis and vascular response in COVID-19. Our results revealed that blood coagulation, platelet activation and aggregation in the control group may be characterized by low expression of genes controlling the above processes (Figure 3). On the other hand, on day 0, the SARS-CoV-2 positive group develops a hypercoagulable state. This is manifested by peak upregulation of coagulation, platelet activation and aggregation. In the subsequent days, these genes and processes tend to downregulate. On day 14, the coagulation activity is comparable to the control group. It is very well established that COVID-19 is associated with hypercoagulability and leads to many thrombotic complications. Klok et al. conducted a retrospective multicenter study that analyzed data from 184 ICU patients with confirmed COVID-19 pneumonia. Their results showed a high rate of thrombotic complications—31% (95%CI 20–41%). More precisely, venous thromboembolism was confirmed in 27% (95%CI 17–37%) and arterial thrombotic events in 3.7% (95%CI 0–8.2%) [61]. Middeldorp et al. investigated the relationship between the severity of the disease and the incidence of thrombotic complications. They demonstrated that patients admitted to the ICU more often suffered from thrombotic events than those who did not require admission to the ICU [62].

Subsequently, we demonstrated that in SARS-CoV-2 positive patients, genes and processes involved in the regulation of angiogenesis were typically downregulated compared to healthy controls (Figure 4). On the other hand, we demonstrated VEGFR upregulation, implying greater activity of angiogenesis-related actions. It can be hypothesized that after the initial release of angiogenic factors, genes are rapidly downregulated as a result of negative feedback. Moreover, the results of other studies have shown increased angiogenic activity. Ackermann and colleagues analyzed lung tissue obtained from autopsies from COVID-19, influenza, and age-matched, uninfected control lungs. They demonstrated an increase in both intussusceptive and sprouting angiogenesis in COVID-19 patients. In addition, the degree of angiogenesis positively correlated with the duration of hospitalization [63]. Similar results were obtained by Pérez-Mies et al. who showed that vascular proliferation is a common finding in the lungs of patients with severe COVID-19 [64]. Nitric oxide (NO) mediates, among others, in vasodilation and exhibits numerous protective properties for endothelial cells [65]. Moreover, NO exhibits antiviral activity [66], including the inhibition of SARS-CoV-2 replication [67]. Figure 5 depicts that processes associated with NO generation are downregulated in COVID-19 patents compared to healthy controls. Therefore, it can be hypothesized that the endothelium in SARS-CoV-2 infection is more susceptible to damage. Indeed, NO generation and bioavailability have been shown to be impaired in severe disease. The authors concluded that endothelial oxidative stress with the resulting decreased bioavailability of NO appears to be a likely pathogenic factor of endothelial dysfunction in ICU patients with COVID-19 [65]. Moreover, it was postulated that NO can potentially be implemented for the treatment of COVID-19 [68]. Interestingly, according to Gelzo et al. expression of inducible nitric oxide synthase (iNOS), the main source of NO during infection, differed between COVID-19 waves. They revealed that in the first wave, the serum levels of iNOS, IL-6, IL-10 increased significantly, according to the severity of the World Health Organization (WHO) scoring, while in the second wave, iNOS did not differ. The second wave patients showed lower levels of iNOS, IL-6, and IL-10 compared to the corresponding first wave subgroup. It should be noted that the researchers showed an increase in iNOS activity, which contradicts our results to some extent [69]. It can be concluded that NO metabolism, in the course of COVID-19 is multifaceted, and its in-depth understanding requires further studies.

In this paper, we demonstrated that vWF/ADAMTS 13 imbalance, which may be a driver of thrombotic complications, is associated with severe disease. Additionally, increased plasma concentration of VEGFR may be a potential predictor of ICU admission. In parallel, low plasma DPP-IV levels may also be a predictor of severe disease. Although these findings shed more light on the pathophysiology of the severe infection, the precise underlying mechanisms have not been fully revealed. Several putative explanations can be hypothesized. For instance, single nucleotide polymorphisms (SNPs) in genes encoding pro-inflammatory cytokines, such as Il-1 or Il-6, or immunomodulatory cytokines, such as Il-10, may be a causative event that triggers subsequent events. Another potential explanation may be uncontrolled overactivity of immune cells mediating the inflammatory response. The above phenomena are purely speculative and may therefore constitute a future direction of research. In addition to the background underlying severe disease, an interesting and clinically useful direction of research is the long-term complications of SARS-CoV-2 infection

## 4. Materials and Methods

### 4.1. Study Group

A retrospective cohort study was conducted on 210 patients diagnosed with COVID-19 at the Department of Infectious, Tropical Diseases and Acquired Immunodeficiency, Pomeranian Medical University in Szczecin, Poland. Standard nasopharyngeal swabs were used to confirm SARS-CoV-2 infection using the real-time polymerase chain reaction (RT–PCR) technique. The control group included 80 healthy hospital employees with negative nasopharyngeal swabs and negative ELISA test results for IgG, IgM, and IgA antibodies specific for SARS-CoV-2. Blood was collected from both groups participating in the study to determine the expressions of a predefined group of genes determining specific vascular processes and the concentration of selected vasoactive factors. All participants were asked to fill out in-depth questionnaires about their general health. The Ethics Committee of the Pomeranian Medical University in Szczecin consented to the study (KB-0012/83/2020), which was conducted in accordance with the Declaration of Helsinki. Prior to enrollment in the study, each participant signed an informed consent form. More details can be found in our previous article [23].

### 4.2. General Health Questionnaire

All patients were interviewed and underwent a physical examination to gather information on the presence of signs and symptoms such as fever, dyspnea, cough, cold, sore throat, fatigue, chest pain, smell/taste abnormalities, headache, body pain, or diarrhea, as well as the severity and duration of the above symptoms. Information about laboratory test results, the need for oxygen or respiratory therapy, the presence of pneumonia on chest computed tomography (CT), the need for hemodialysis, and the patient’s death were collected from electronic medical records. At the time of study entry, all demographic information, family history, history of chronic disease, and other general health risk factors were recorded. COVID-19 severity was retrospectively determined. All patients included in the study who tested positive for COVID-19 were divided into two groups depending on the severity of their disease. Group 1 (patients in the intensive care unit—ICU) included individuals who needed to be admitted to the ICU. Group 2 (non-ICU patients) included asymptomatic or mildly symptomatic patients who did not require hospitalization due to COVID-19 and symptomatic patients with an oxygen saturation below 95% who did not need admission to the ICU. For more details please refer to our previous paper [23].

### 4.3. Plasma Collection

Peripheral blood (PB) samples were taken on day 1 (hospital admission) and again during hospitalization/isolation on days 7, 14, and 28 after the COVID-19 diagnosis. PB samples (~7.5 mL) were centrifuged after being collected in EDTA tubes (2000 rpm, 10 min). The plasma was then transferred to a new tube and centrifuged once more under the same conditions. Plasma samples were kept at −80 °C. To obtain blood mononuclear cells, plasma-free blood was lysed with Lysing Solution (BD Biosciences, San Jose, CA, USA) for 15 min at room temperature. For more details please refer to our previous paper [23].

### 4.4. RNA Isolation

Viral RNA was extracted using the MagMAX Viral/Pathogen II Nucleic Acid Isolation Kit (Thermo Fisher Scientific, Markham, ON, Canada) according to the manufacturer’s instructions. Three processing plates (KingFisher 96 Deep-Well Plate, Thermo Fisher Scientific, Markham, ON, Canada) with Wash 1 Solution (500 µL per well), Wash 2 Solution—80% ethanol (1000 µL per well), and Elution Solution (50 µL per well) were also made. Using an automated KingFisher Flex equipment (Thermo Fisher Scientific, Markham, ON, Canada), MagMAX Viral/Pathogen nucleic acid extraction was carried out. Total RNA was extracted from blood mononuclear cells using the commercial mirVanaTM miRNA Isolation Kit (Thermo Fisher Scientific, Markham, ON, Canada). The extraction was carried out according to the manufacturer’s instructions. An Epoch spectrophotometer determined the final concentration. For more details please refer to our previous paper [23].

### 4.5. qRT–PCR Assays for Detecting SARS-CoV-2 RNA

For the detection of SARS-CoV-2 RNA, qRT-PCR assays were carried out using a QuantStudio 5 PCR instrument and a TaqPath COVID 19 CE IVD RT PCR Kit (Thermo Fisher Scientific, Markham, ON, Canada), in accordance with the manufacturer’s protocols. Following RT–PCR completion, results were analyzed using Applied Biosystems COVID-19 Interpretive Software v1.5.1. (Thermo Fisher Scientific, Markham, ON, Canada). Tests were deemed positive if at least 2 out of the 3 analyzed SARS-CoV-2 genes (ORF1ab, N, S) had Ct values ≤ 37. For more details please refer to our previous paper [23].

### 4.6. Affymetrix GeneChip Microarray Expression Study

The GeneChip WT PLUS Reagent Kit (Affymetrix, Santa Clara, CA, USA) was utilized to prepare RNA for hybridization. The detailed procedure has been described previously [70,71]. Briefly, a two-step cDNA synthesis reaction was performed. The cDNA was then biotin-labeled and fragmented using the Affymetrix GeneChip WT Terminal Labeling and Hybridization kit (Affymetrix, Santa Clara, CA, USA). cDNA fragments were hybridized to the Affymetrix Human Gene 2.1 ST ArrayStrip (20 h, 48 °C). The microarrays were then stained using the Affymetrix GeneAtlas Fluidics Station (Affymetrix, Santa Clara, CA, USA). The array strips were scanned with the Imaging Station of GeneAtlas System (Thermo Fisher Scientific, Waltham, ON, Canada). The Affymetrix GeneAtlas TM Operating Software v.1.0.6. (Affymetrix, Santa Clara, CA, USA) was used for preliminary analysis of the scanned chips. For more details please refer to our previous paper [23]. 

### 4.7. Assignment of Differentially Expressed Genes to Relevant Gene Ontology Biological Process (GO BP) Terms

The DAVID (Database for Annotation, Visualization, and Integrated Discovery) bioinformatics tool was employed [72]. All gene IDs of differentially expressed genes were uploaded to DAVID using the “RDAVIDWebService” BioConductor library [73]. Differentially expressed genes from each comparison were visualized as a heatmap using the “ComplexHeatmap” library [74]. For more details please refer to our previous paper [23].

### 4.8. Luminex Assay

Plasma levels of selected vascular factors, including ADAMTS13, Angiopoietin-2, DPPIV/CD26, EGF, Endothelin-1, E-Selectin/CD62E, FGF acidic/FGF1, Flt-3 Ligand/FLT3L, PDGF-AA, PDGF-AB, Serpin E1/PAI-1, Thrombomodulin/BDCA-3, VEGF, VEGFR1/Flt-1, and vWF-A2, were quantified using multiplex fluorescent bead-based immunoassays (Luminex Corporation, Austin, TX, USA) with a commercial R&D Systems Luminex Human Discovery Assay (R&D Systems, Minneapolis, MN, USA). A volume of 50 µL of blanks, standards, and samples was added to the plate along with the Microparticle Cocktail and incubated in the dark for 2 h at room temperature on a horizontal orbital microplate shaker. For more details please refer to our previous paper [23].

### 4.9. Statistical Analysis

Quantitative data were expressed as median and interquartile range (IQR). The Shapiro-Wilk test was used to determine the distribution of quantitative data. Due to the fact that continuous variables deviated from normality, the Mann-Whitney U test was implemented to assess differences between the non-ICU and ICU groups. *p* < 0.05 was considered statistically significant. All calculations were performed in R [75]. 

## 5. Conclusions

Severe COVID-19 is associated with vWF/ADAMTS 13 imbalance, which may contribute to thrombotic complications. A higher plasma concentration of VEGFR may be a potential predictor of ICU admission. On the other hand, a low plasma concentration of DPP-IV also predicts severe disease. SARS-CoV-2 infection impairs angiogenesis, hinders the generation of nitric oxide, and thus impedes vasodilation. In addition, a hypercoagulable state develops mainly in the early stages of the disease, which may contribute to the well-established complications of COVID-19.

## 6. Strengths of the Study

The study was conducted on a large study group (210 patients, 80 healthy volunteers) using modern molecular techniques. Detailed assessment of numerous vascular factors at the protein level allowed us to identify potential predictors of severe COVID-19. At the same time, bioinformatics analysis at the mRNA level allowed us to better understand the pathophysiology of processes related to vascular response, inflammation and angiogenesis. The obtained results contributed to the formulation of further research hypotheses and will become the basis for conducting further, more cross-sectional studies taking into account other factors not included in the current study

## 7. Study Limitations

Our study revealed interesting findings and extended the current understanding of the pathophysiology of severe COVID-19. Despite its strengths, our study has some minor drawbacks. First, we measured the plasma levels of various vascular factors. However, we did not measure plasma levels of the RNA encoding these proteins. Such an analysis would provide a broader insight into the pathophysiology of severe COVID-19. Next, we isolated RNA from blood mononuclear cells and performed a thorough bioinformatics analysis. Nevertheless, we have not focused on epigenetic mechanisms, which are worth exploring and may additionally reveal novel mechanisms underlying the severity of the disease.

## Data Availability

Data are available upon request from the correspondence authors.
